# Predicting the Membrane Permeability of Fentanyl and Its Analogues by Molecular Dynamics Simulations

**DOI:** 10.1021/acs.jpcb.1c05438

**Published:** 2021-07-21

**Authors:** Christopher Faulkner, Nora H. de Leeuw

**Affiliations:** † School of Chemistry, Cardiff University, Main Building, Park Place, Cardiff CF10 3AT, U.K.; ‡ School of Chemistry, University of Leeds, Leeds LS2 9JT, U.K.

## Abstract

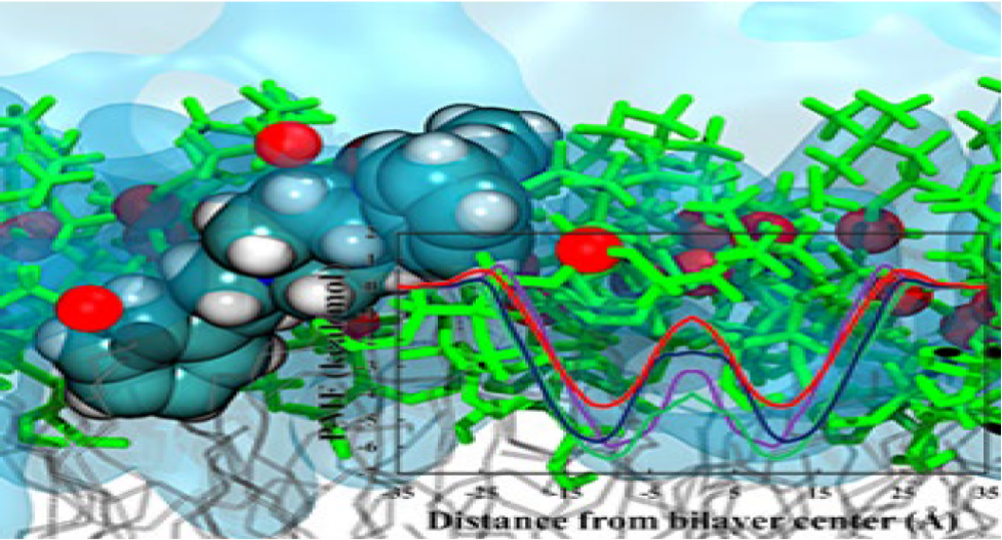

The lipid membrane is considered a crucial component of opioid general anesthesia. The main drug used for the induction and maintenance of opioid anesthesia is fentanyl and its various analogues. However, these drugs have different clinical effects, and detailed atomic-level insight into the drug–membrane interactions could lead to a better understanding how these drugs exert their anesthetic properties. In this study, we have used extensive umbrella sampling molecular dynamics simulations to study the permeation process of fentanyl and three of its analogues into a variety of simple phospholipid membrane models. Our simulations show that we can accurately predict the permeability coefficients of these drug molecules, which is an important process in understanding how pharmaceuticals reach their molecular targets. We were also able to show that one phospholipid provides more accurate predictions than other lipids commonly used in these types of permeation studies, which will aid future studies of these types of processes.

## 1 Introduction

The discovery of opioid molecules, for example, morphine, that produce effects such as the desensitization to painful stimuli, which is thought to be caused by binding to, and modulation of, G-protein-coupled receptors, has contributed significantly to the advance of modern medicine and surgical procedures. Fentanyl is an opioid analgesic/anesthetic that is thought to be at least 80 times more potent than morphine,^1^ which has made fentanyl one of the most used opioids in the general anesthesia process and a prominent drug of abuse.^2^ Despite the highly addictive nature of this drug, fentanyl and its various analogues are routinely used in surgical procedures, owing to their rapid onset times, duration of action, potentiation of general anesthetics, and their excellent ability to effectively desensitize the patient to painful stimuli. Fentanyl-based opioids have interesting properties, so they can be used as partial or complete general anesthetics.^3,4^

To date, there is no widely accepted method by which these drugs exert their anesthetic effect, even though it has been related to both binding with various membrane proteins^5,6^ and possible effects on the lipid membranes, as is commonly associated with inhalational anesthetics.^7,8^ There is strong evidence, put forward by Stone et al,^9^ that the lipid membrane is crucial in the process to achieve opioid anesthesia. Their findings show a relation between the calculated brain lipid membrane concentrations of opiates with a defined minimum alveolar concentration (MAC) and electroencephalographic changes, which strongly suggests a lipid membrane site for the anesthetic action of opiates, at least up to the 50% MAC reduction level.^9^ The correlation shown between the anesthetic effects and the membrane lipid component, as opposed to serum opioid levels, highlights the importance of studying opioid–lipid interactions. The lipid membrane has also been shown to be crucial for fentanyl binding to membrane proteins; because of the high lipophilicity of fentanyl and its analogues, the lipid membrane can act as a route for the drugs to bind to transmembrane binding sites.^10,11^

**Figure 1:**
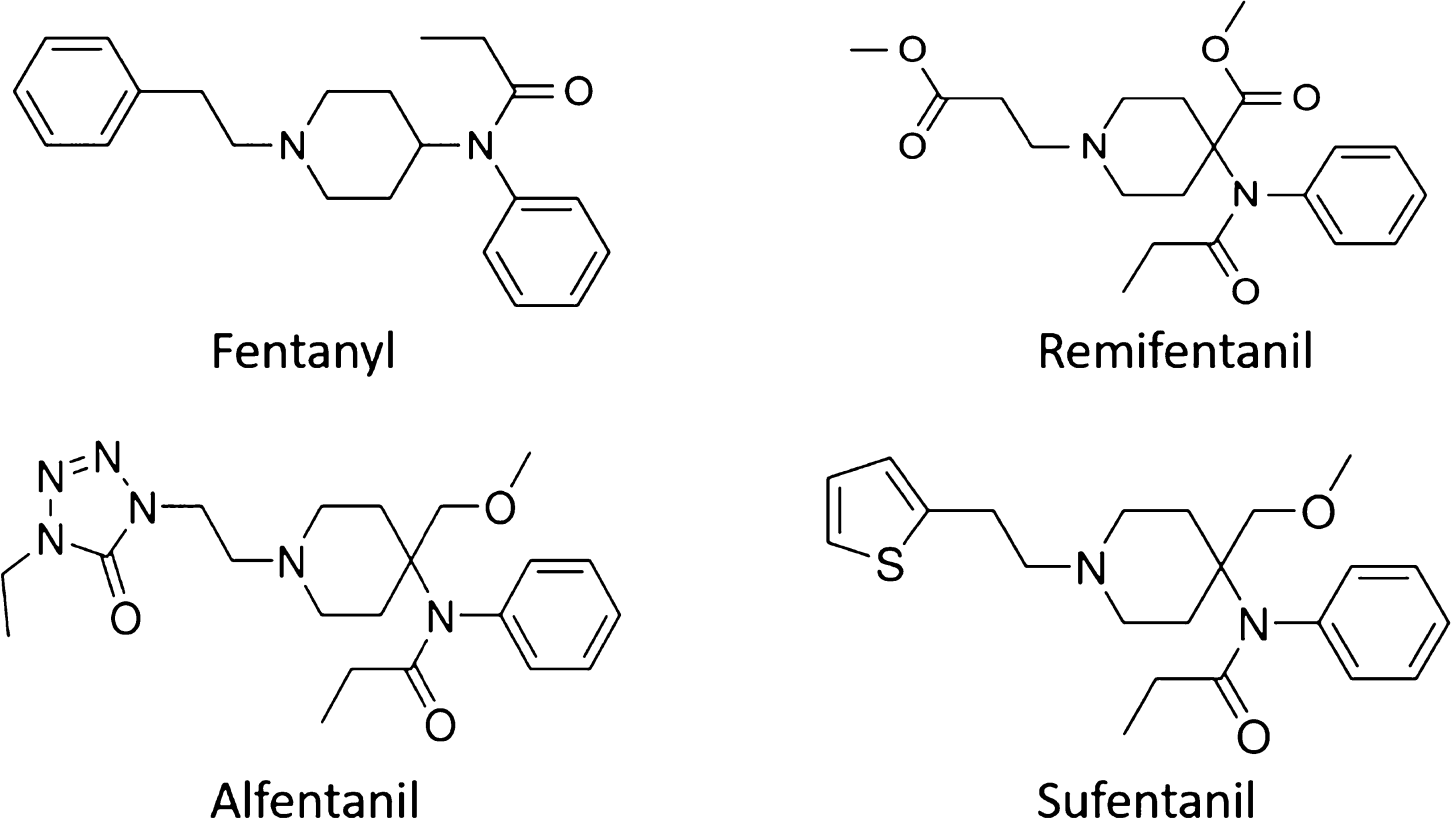
Chemical structures of the four opioid drugs studied.

The lipid membrane is a crucial component of the cell, which acts as a barrier to passive diffusion of small molecules and ions, although many small molecules, such as pharmaceuticals, can permeate through the lipid membrane, depending on its composition and the properties of the solute. Fentanyl and its analogues are lipophilic in nature, so they are expected to be able to easily enter the membrane environment. In this paper, we study four fentanyl-based opioid drugs, that is, fentanyl, alfentanil, remifentanil, and sufentanil (Figure 1) in four model membrane bilayer systems, namely, 1,2-dimyristoyl-sn-glycero-3-phosphocholine (DMPC), 1-palmitoyl-2-oleoyl-glycero-3-phosphocholine (POPC), 1,2-dioleoyl-sn-glycero-3-phosphocholine (DOPC), and 1,2-dipalmitoyl-sn-glycero-3-phosphocholine (DPPC). These different bilayers have a diverse range of lipid tails with differing chain lengths and saturation, which will provide insights into any changes in the permeabilities of these drugs in different parts of the cellular membrane.

Investigations into the permeation of drug molecules into the cell membrane are highly important to understand and achieve the delivery of drug molecules to their molecular targets. Many experimental techniques have been developed to investigate this important property, such as cell-based CaCo-2 assay^12^ and parallel artificial membrane permeability assay (PAMPA).^13^ These methods are widely used in industry and academia to calculate the permeabilities of various types of compounds, but they provide no information on the biophysics of membrane permeation.^14^ Various linear response models and mathematical models, such as the quantitative structure permeability relationship^15^ and steady-state models,^16^ have been developed to make predictions based on experimental test sets, but their predictive performance has been relatively poor, and atomistic details of the processes of permeation cannot be deduced.^17^ To gain atomic-level insight into the passive permeation of fentanyl and its analogues (shown in Figure 1), we have employed atomistic molecular dynamics (MD) simulations in combination with an umbrella sampling technique and the weighted histogram analysis method (WHAM) to construct the potential of mean force (PMF) curves for the drug permeation. When trying to predict properties such as permeability, many previous studies use only one phospholipid system, such as DPPC,^18^ but we were interested in comparing a number of phospholipid systems, as well as a variation of fentanyl-type molecules. This study has two main goals: First we wished to ascertain whether MD simulations using the umbrella sampling method can accurately predict permeability coefficients for fentanyl and its analogues using simple bilayer models and second, we wished to determine if the phospholipid used in the model makes a difference to the permeability predictions and what lipid is most reliable for simulation of such systems.

## 2 Computational Details

### 2.1 System Preparation and Simulation Setup

All model membrane bilayers were constructed using the CHARMM-GUI membrane builder.^19^ Each system consisted of 64 lipids per leaflet with a water buffer of 35 Å on either side. All of the simulations used the TIP3P water model^20^ and the lipid14 parameters^21^ for the lipids. The drug molecule parameters were generated using the antechamber program^22^ with the AM1-BCC charge model and the GAFF2 forcefield.^23^ Pure membrane systems containing no drug molecules were initially minimized and equilibrated in multiple stages. In stage 1, the system was minimized for 10,000 steps using the steepest descent method, then 10,000 steps using the conjugate gradient method. The systems were then heated in two stages, with the first stage heating the system to 100 K using a Langevin thermostat^24^ with a 10 kcal/(mol Å^2^) harmonic restraint applied to the lipid molecules. The second phase slowly heated the system to the desired production temperature (303 K for DOPC, POPC, and DMPC and 323 K for DPPC) for 100 ps. Anisotropic Berendsen pressure regulation was introduced here to control the pressure at 1 atm, in addition to the temperature control provided by the Langevin thermostat. The same restraints were applied to the lipid molecules. The final stage of the equilibration involved slowly reducing the harmonic restraints of the lipid molecules over 10 ns of NPT simulation. 225 ns production runs were then carried out on all the pure membranes, which were then analyzed to confirm if the models were in the correct, biologically relevant Lα phase. Analysis was carried out using cpptraj^25^ and in-house scripts (results shown in Supporting Information Section 1) and compared to experimental data. The first 25 ns of the simulation of the pure membrane systems was discarded, and analysis was performed on the last 200 ns of the trajectories. All simulations in this paper were carried out using the GPU implementation of the AMBER18 code.^26^ Three-dimensional periodic boundary conditions were used with the usual minimum image convention, and the SHAKE algorithm^27^ was used to constrain bonds involving hydrogen allowing for a 2 fs timestep. PME was used with a cutoff of 10 Å to treat the electrostatic interactions, and a long-range analytical dispersion correction was applied to the pressure and energy. A collision frequency of γ = 1.0 ps^–1^ was used for the Langevin thermostat, and a pressure relaxation time of 1.0 ps was used for the anisotropic Berendsen barostat (1 atm). For the drug molecule simulations, the drug was added to the center of the bilayer, and a harmonic restraint of 10 kcal/(mol Å^2^) was applied. A 10 ns simulation was carried out to equilibrate the lipid with the drug molecule present. Steered MD simulations were used to pull each drug molecule through each bilayer into the water phase at a speed of 1.0 Å/ns (35 ns for each drug in each system). Coordinates of the system with the drug molecule at equally spaced locations over the pathway were extracted and used as starting states for the umbrella sampling simulations. Full profiles were obtained by symmetrizing the data as we are not using asymmetric or multicomponent bilayers, where it would be important to pull in both directions to study the differences between each leaflet.

### 2.2 Umbrella Sampling Simulations

The reaction coordinate for the drug permeation was defined as the z-component of the distance between the center of mass of the lipid nitrogen atoms and the heavy atoms in the drug molecule. For each drug in each bilayer, a total of 35 windows separated by 1.0 Å were used with a biasing harmonic restraint of 2.5 kcal/(mol Å^2^) using the AMBER umbrella COM restraint code. Each window for each drug molecule was simulated for 100 ns, totaling 3.5 μs of sampling per drug molecule per bilayer, which totals 56 μs of sampling for all systems. The probability distributions obtained from these simulations were reweighted using the WHAM (histograms shown in Supporting Information Section 2).^28^ The local diffusivity for each window was estimated using the Hummer positional autocorrelation extension to the Woolf–Roux estimator^29^
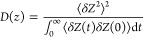
where δ*Z*(*t*) = *z*(*t*)–⟨*Z*⟩ and *Z* is a position on the *z*-axis and *z*(*t*) is a position on the *z*-axis at time *t*. The obtained PMF and *D*(*Z*) values were interpolated at 1.0 Å intervals, and the results were used to calculate the resistivity (*R*) and permeability (*P*) by:
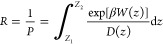
where β = 1/*k*_B_*T* and *z* is the position of the drug molecule along the transmembrane axis, and *W*(*z*) is the PMF. The lower and upper integration bounds are points in the center of the membrane and water phase.

Umbrella sampling histograms were unbiased by the WHAM with 720 bins and a tolerance of 1 × 10^–8^ for window offsets. The statistical uncertainty at each bin was estimated using bootstrapping, with 100 bootstrap trials for each PMF.

## 3 Results and Discussion

**Figure 2:**
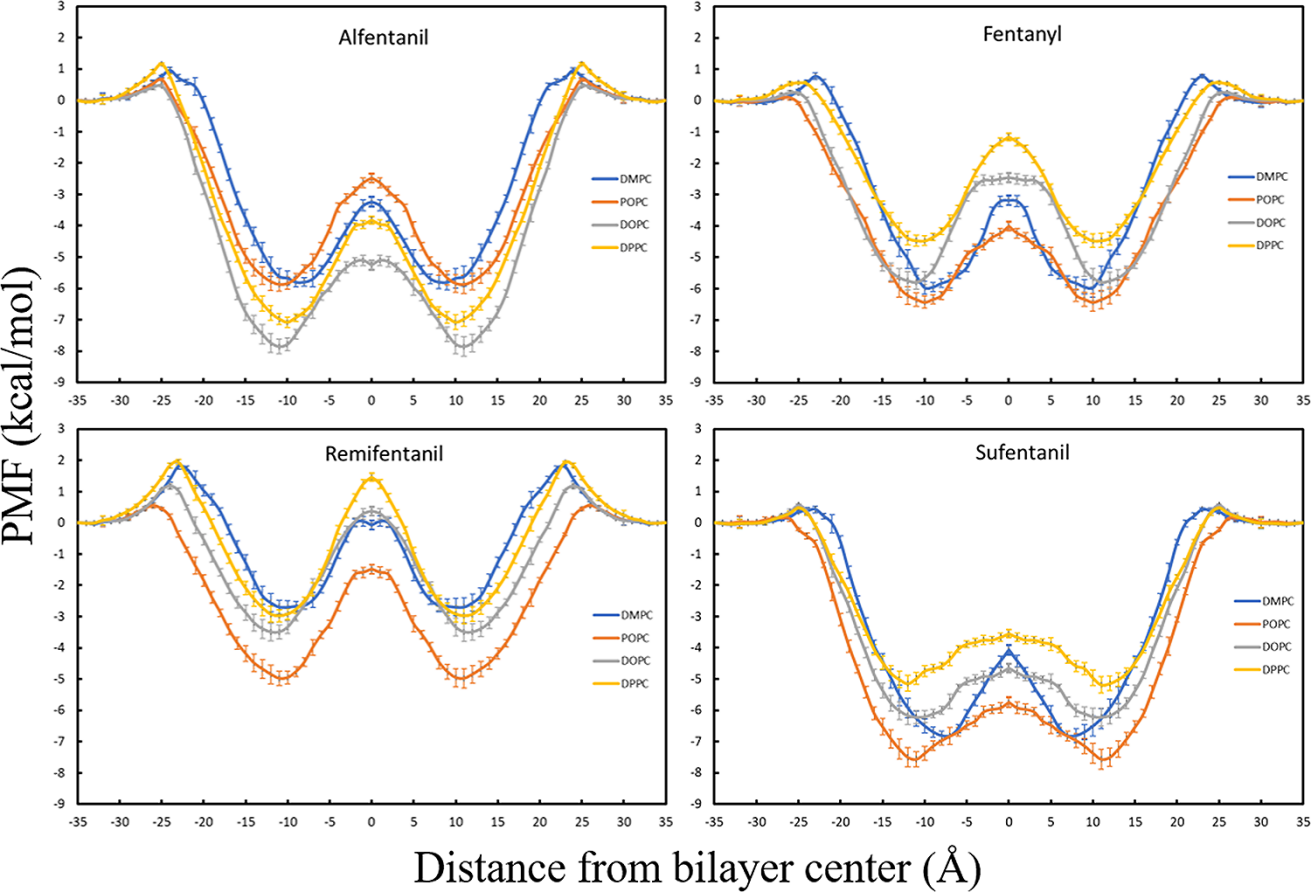
Free energy profiles calculated for all permeating molecules and bilayers.

The permeation free energy profiles for the selected four fentanyl molecules, simulated in DOPC, POPC, DPPC, and DMPC, are shown in Figure 2. These opioid molecules are all hydrophobic to varying extents, but the PMF profiles show expected behavior for these types of drug molecules. There is a small positive energetic barrier—largest at 1.94 ± 0.10 kcal/mol for remifentanil in DPPC—when the drugs permeate into the hydrophilic phosphatidylcholine headgroup followed by a global minimum in the bilayer interior with the lowest being −7.86 ± 0.24 kcal/mol for alfentanil in DOPC.

To rationalize the position of the minima in our PMF profiles, we can use the four-region model, which has been described in previous publications.^30,31^ In this model, region three from 6 to 13 Å from the bilayer center is the high-tail density region. Hydrophobic molecules will have many favorable hydrophobic interactions with the lipid tails in this region, so the difference in free energy here can be explained by the greater number of contacts between the drug molecules and the lipid tails. The barriers observed at the center of the bilayers for all drug molecules could be a consequence of entropic factors, such as a reduction in the lipid tail mobility when the drug molecule is present. However, the more likely explanation is the decrease in the drug–lipid interactions at the center, because of the lower density of atoms in this region, where the lower interaction energies would therefore disfavor the presence of the drug molecule in this region. This behavior has been shown to be the main factor for the local anesthetic, benzocaine, and the antiepileptic drug, phenytoin.^32^ The slight variation in the PMF profile shapes between certain drug/lipid combinations is due to the diversity in the structure and properties of each drug molecule and the differences in the structure of the lipid chains. The different packing arrangements and dynamics of the chains will lead to slightly different interactions with each drug molecule and hence slight changes in the PMF shape.

**Figure 3:**
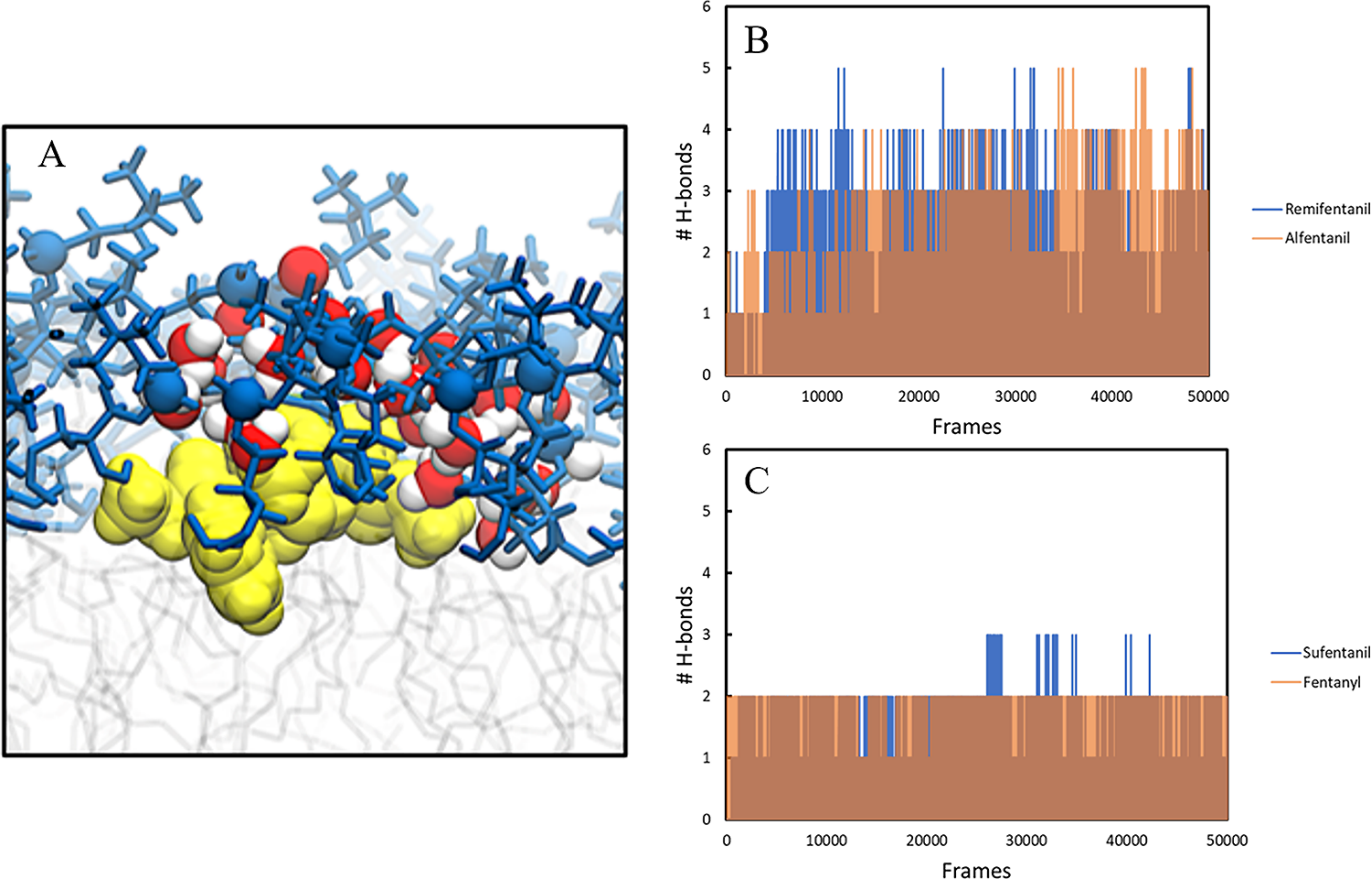
(A) Water molecules solvating remifentanil (yellow) at the head group/hydrophobic interface (blue). (B) Hydrogen-bond plot for remifentanil and alfentanil. (C) Hydrogen-bond plot for sufentanil and fentanyl. Hydrogen-bonding data were calculated in the 21–25 Å windows, where energy barriers were observed and averaged for each drug. Transparency has been added to alfentanil and fentanyl to improve visibility.

We can see from our PMF profiles (Figure 2) that even though all the bilayers have the same PC headgroup, they have different tails, which clearly alter the permeation process. From a visual inspection of the umbrella sampling windows from the different bilayer systems, we observe clear differences in the mobility of the head groups because of the different lipid chains, which causes local rearrangements to the extent that exposure of the drug molecules to the tail regions varies. We also observe water molecules entering the head group/hydrophobic interface with both alfentanil and remifentanil, which stabilizes these molecules through hydrogen-bonding and accounts for the energetic barriers observed (Figure 3). These two analogues have the most hydrogen-bond acceptor sites, and hence they interact with more water molecules than the other two solutes. Hydrogen-bonding plots (Figure 3B,C) confirm that more water molecules are bound to alfentanil and remifentanil in the head group/hydrophobic interface region, where the data were calculated. We note that the extent of sampling in these simulations is much greater than that in other studies of solute permeation, and we are therefore more likely to observe rearrangements that are not achievable in shorter simulations.^33,34^

**Figure 4:**
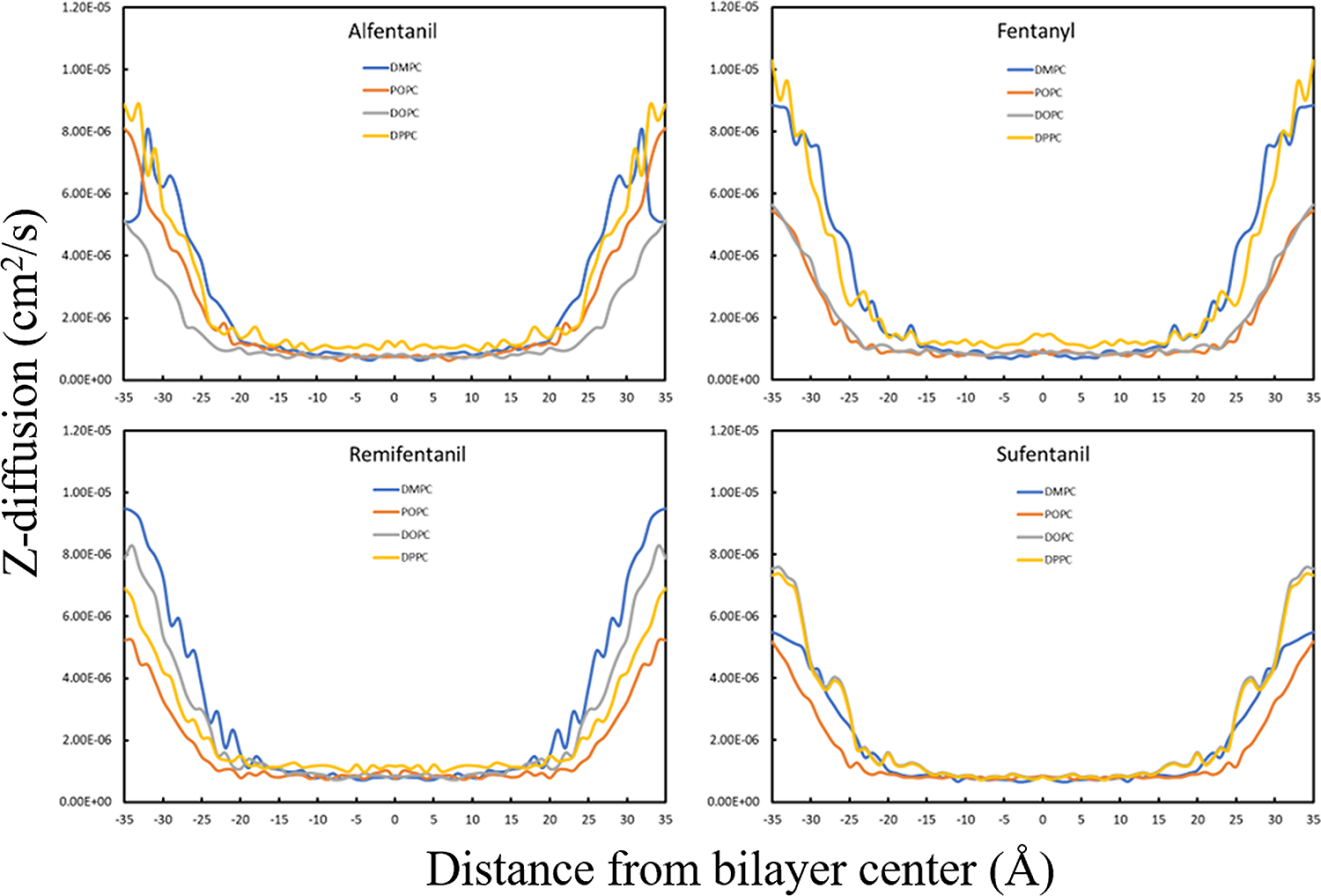
z-diffusion profiles calculated for all permeating molecules and bilayers.

The position-dependent diffusion coefficients for fentanyl and its analogues do not vary significantly from one another (Figure 4). The average diffusion within the hydrophobic core of the bilayer (*z* = −20 to 20 Å) for the drug molecules range from ∼1 × 10^–6^ to ∼2 × 10^–6^ cm^2^/s. Within the bilayer core, the diffusion coefficient values for the drug molecules reach their minimum plateau at *z* ≈ 5–10 Å, but increase slightly as the drug approaches the center (*z* = 0 Å), which is the most disordered area of the bilayer core. All the drugs show an increase in diffusion in the bulk water phase that is close to an order of magnitude greater than the calculated results within the bilayer core. This finding is consistent with previous constrained MD simulations for drug molecules passing through lipid bilayers,^35^ and the results obtained for our four drug molecules are therefore as expected for lipophilic compounds.

**Figure 5:**
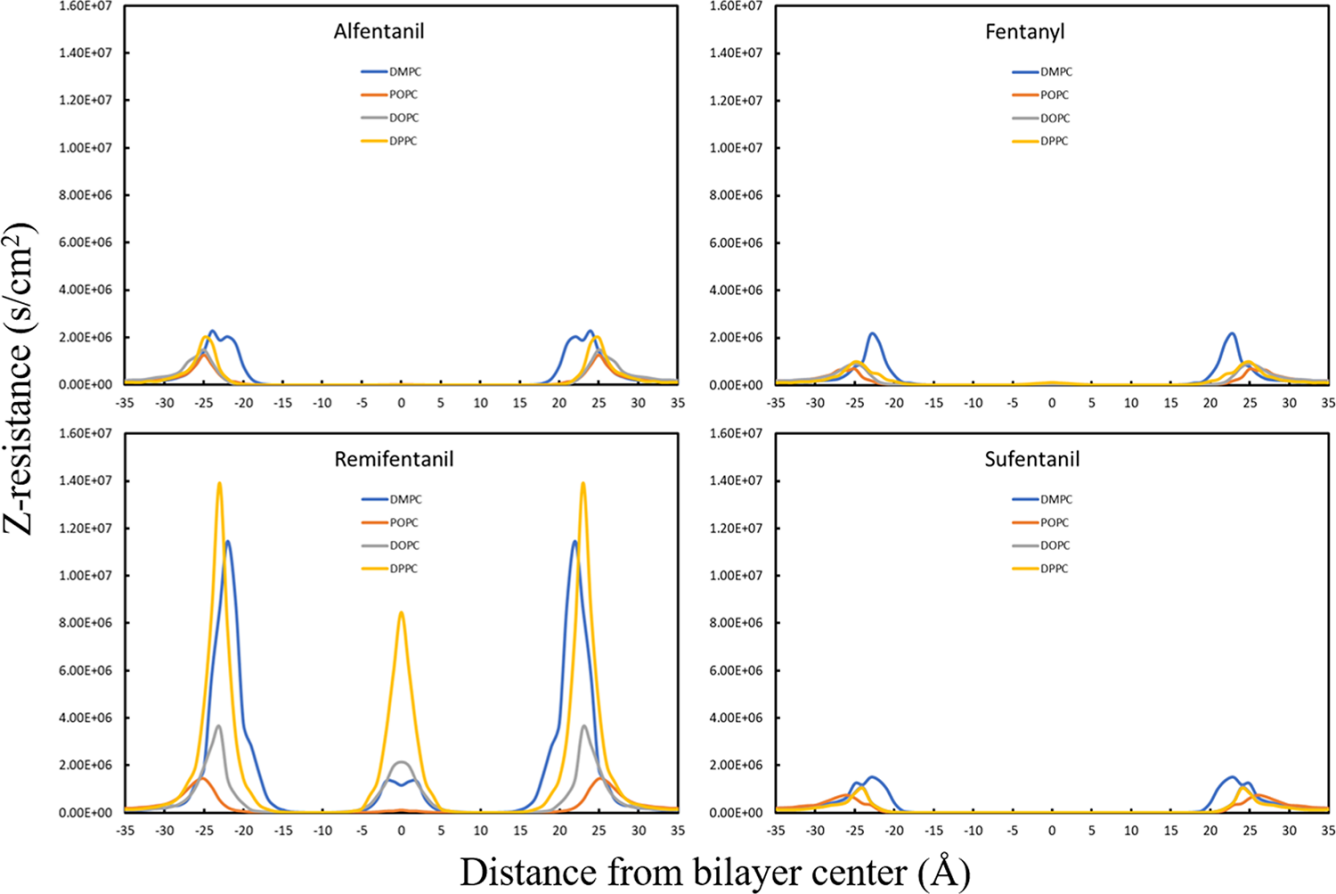
Resistance to permeation calculated for each drug molecule in each bilayer.

Profiles for the resistance to permeation for each drug molecule are shown in Figure 5. Fentanyl and its analogues are lipophilic drug molecules, and we would therefore expect the largest resistance to permeation to occur at the lipid–water interface, which is indeed observed. The lipid head group region is partially charged and polar, which for hydrophobic molecules offers the largest resistance to permeation. The resistance plots follow the free energy profiles for all molecules, shown particularly clearly for remifentanil. This agreement is expected, and its occurrence is therefore important for the validation of our calculations. The resistance increases steeply as the molecules pass through the head group region and again increases slightly at the disordered bilayer center, indicating that the resistance is dominated by the free energy component. This behavior shows that higher free energy contributions lead to higher resistance to permeation for fentanyl-based opioids.

The calculated permeability coefficients from our simulations are presented in Table 1 along with experimentally determined permeability coefficients from a variety of different experimental techniques.

**Table 1:** Calculated and Experimentally Determined Permeability Coefficients for Fentanyl and the Analogues Studied

drug	calculated permeability coefficient (cm/s)	experimental permeability coefficient (cm/s)
alfentanil	–6.49 (DOPC)	–0.06 (porcine polar brain lipid)^36^
	–6.15 (DPPC)	–2.11 (microvessel lipid)^36^
	–8.35 (POPC)	–2.88 (microvessel lipid + cholesterol)^36^
	–3.73 (DMPC)	–1.75 (dodecane)^36^
		–4.42 (spinal meninges)^37^
		–3.53 (PAMPA)^38^
		–3.49 (Caco-2)^38^
		–3.54 (Caco-2/MDCK)^39^
fentanyl	–8.67 (DOPC)	–2.13 (porcine polar brain lipid)^36^
	–8.12 (DPPC)	–2.48 (microvessel lipid)^36^
	–10.94 (POPC)	–3.60 (microvessel lipid + cholesterol)^36^
	–5.42 (DMPC)	–4.81 (spinal meninges)^37^
		–4.32 (BBB-PAMPA)^40^
		–5.81 (human skin)^41^
		–6.16 (PAMPA)^42^
		–4.89 (human skin)^43^
		–3.22 (BBB-PAMPA)^39^
sufentanil	–10.09 (DOPC)	–3.15 (porcine polar brain lipid)^36^
	–8.09 (DPPC)	–2.69 (microvessel lipid)^36^
	–9.33 (POPC)	–2.78 (microvessel lipid + cholesterol)^36^
	–5.00 (DMPC)	–3.57 (dodecane)^36^
		–4.90 (spinal meninges)^37^
		–5.48 (human skin)^41^
		–4.84 (human skin)^43^
		–3.87 (BBB-PAMPA)^44^
remifentanil	–2.49 (DOPC)	–0.33 (porcine polar brain lipid)^36^
	–0.80 (DPPC)	–2.49 (microvessel lipid)^36^
	–5.81 (POPC)	–2.40 (microvessel lipid + cholesterol)^36^
	–0.93 (DMPC)	–3.76 (dodecane)^36^

It is clear from our calculated results that the umbrella sampling method can predict the correct trend in permeability coefficients for these drug molecules. The main experimental methods used in industrial and pharmaceutical research to obtain permeability coefficients for drug molecules are PAMPA, BBB-PAMPA (blood–brain barrier), and Caco-2 methods, and the data in Table 1 show that our simulated results compare very well with the available experimental data for alfentanil (−3.73 (DMPC), −3.53 (PAMPA), −3.49 (Caco-2), −3.54 (Caco-2/MDCK)), fentanyl (−5.42 (DMPC), −4.32 (BBB-PAMPA), −6.16 (PAMPA)), and sufentanil (−5.00 (DMPC), −3.87 (BBB-PAMPA)). The results for remifentanil agree less well with the small amount of experimental data available, because of the uncertainty in the charge state of the drug as a result of its susceptibility to ester hydrolysis. Remifentanil was modeled in its neutral phase, which is its expected state in the studies for which experimental data are available. We also note good comparisons between our simulations and other experimental data, for example, from spinal meninges and human skin. This is unexpected, as models for these systems would usually include multiple different lipids with varying cholesterol concentrations, whereas we have achieved good correlation with simple bilayer models but with large amounts of sampling. It has been shown previously that poor sampling leads to inaccurate free energies, which can lead to an order of magnitude of difference in permeability owing to exponential dependence.^45,46^ Upon the addition of cholesterol, we would expect that the lipid tails would become highly ordered because of the favorable van der Waals interactions that would be formed between the cholesterol and the other lipid tails. Thus, when the drug molecules are added, strong van der Waals contacts would be broken, leading to large voids around the drug molecules. A previous experimental study^47^ has shown that the incorporation of cholesterol into a DMPC bilayer increased the hydrophobicity of the bilayer center, hence causing a large hydrophobic barrier to the permeation of polar molecules. With regard to opioid molecules which are hydrophobic in nature, high cholesterol concentrations could increase the barrier significantly and decrease their permeability, which has been shown to occur for other hydrophobic drugs.^48^ Good spinal meninges and human skin comparisons are important for opioid research, as these drugs are often administered as epidural anesthetics and through transdermal patches as analgesics for various chronic conditions. The differences in permeability obtained in each different bilayer show that the basic structural and dynamic properties of simple model bilayers can have a significant impact on the membrane permeability of these drug molecules. The bilayers with the shorter, saturated tails gave better comparisons, which suggests that the higher lipid tail packing in saturated lipids is important for drug permeability. Our results also show that the DMPC bilayer consistently gives the best comparison to the experimental results for our opioid drug molecules, which is an important observation as many studies only use one model bilayer to make predictions, which could introduce errors into the obtained results. However, our results suggest that testing of multiple bilayers in simulation studies is a more rigorous procedure and, where possible, should be carried out to find the best model bilayer for any given drug molecules, thereby leading to more consistent results that minimize potential errors when comparing to experimental data.

## 4 Conclusions

In this article, extensive umbrella sampling simulations of fentanyl and three analogues in four different membrane bilayers were performed to calculate their permeability coefficients and determine which bilayer provides the most accurate results compared to experimental data. Our simulations revealed that for all drug molecules the main resistance to permeation was observed at the lipid head group because of its partially charged, polar nature and the hydrophobic nature of the drug molecules. Our simulations were able to identify the DMPC lipid bilayer as the most reliable lipid to use in the simulations of these drug molecules, as the results obtained compared best with experimental data from the PAMPA and Caco-2 experimental methods used in pharmaceutical drug permeability studies. Using the umbrella sampling method, properties such as local resistance, free energy, and diffusion can be calculated for fentanyl-based drugs in atomic-level detail in different regions of the bilayer and produce accurate permeability coefficients. This method could therefore be of importance in the future design of new fentanyl-based analgesic/anesthetic drugs.

## Supporting Information Available

Results of pure bilayer validation simulations, umbrella sampling window histograms, and PMF profiles plotted for each bilayer (PDF)
