# Supplementary material for: Predicting the Membrane Permeability of Fentanyl and Its Analogues by Molecular Dynamics Simulations
Source: J Phys Chem B. 2021 Jul 21;125(30):8443–9. doi: 10.1021/acs.jpcb.1c05438 (PMC8389899; doi:10.1021/acs.jpcb.1c05438)
Supplement: Supplementary file 1 — jp1c05438_si_001.pdf [file jp1c05438_si_001.pdf]

Supporting information: Predicting the Membrane Permeability of Fentanyl and its Analogues by Molecular Dynamics Simulations

*Christopher Faulkner**†* and Nora H. de Leeuw**†,‡

†School of Chemistry, Cardiff University, Main Building, Park Place, CF10 3AT, Cardiff, United Kingdom

‡School of Chemistry, University of Leeds, Leeds, LS2 9JT, United Kingdom

Corresponding Author

*Email: FaulknerC3@cardiff.ac.uk; deleeuwn@cardiff.ac.uk

1. Results for pure membrane validation simulations

1.1 DMPC structural properties

Table S1. Calculated bilayer structural properties for DMPC compared to experimental values.

DMPC	Area per lipid (Å^2)	Area compressibility modulus (mNm^{-1})	Volume per lipid (Å^3)	Thickness (Å)	Lateral lipid diffusion (10^{-8}cm^{2}s^{-1})
Calculated	60.57 ± 0.1	243 ± 11.0	1055 ± 2.1	34.5 ± 0.2	6.2 ± 1.3
Experiment	60.6, 59.9[1]	234[2]	1101[1]	34.4, 35.3[1]	5.95[3]

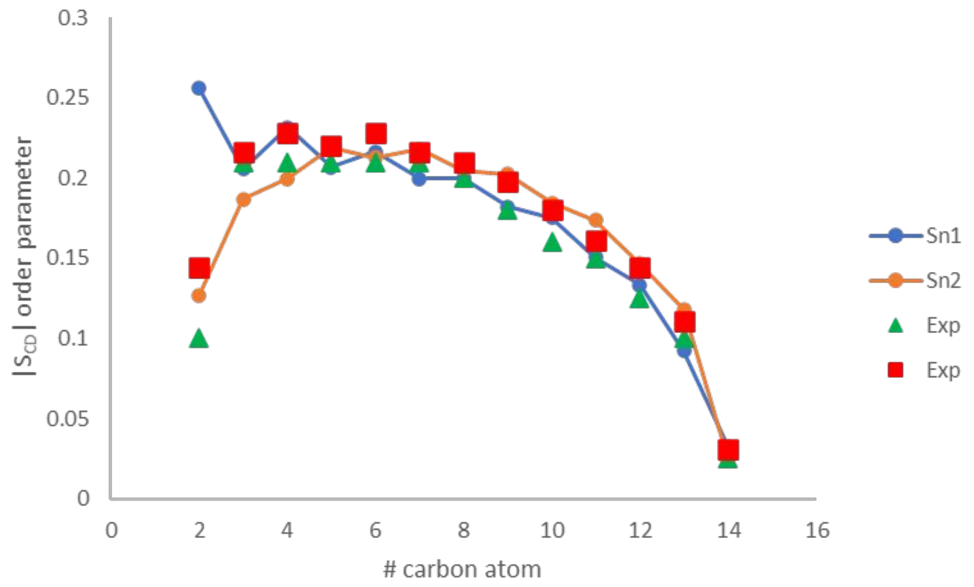

Figure S1. Calculated S_{CD} order parameters for DMPC compared to experimental values.[4,5]

1.2 POPC structural properties

Table S2. Calculated bilayer structural properties for POPC compared to experimental values.

POPC	Area per lipid (Å^2)	Area compressibility modulus (mNm^{-1})	Volume per lipid (Å^3)	Thickness (Å)	Lateral lipid diffusion (10^{-8}cm^{2}s^{-1})
Calculated	65.75 ± 0.2	286.6 ± 16.0	1209 ± 0.6	37.2 ± 0.1	8.5 ± 1.1
Experiment	64.3, 68.3[6]	180-330[7]	1256[6]	37[6]	10.7[8]

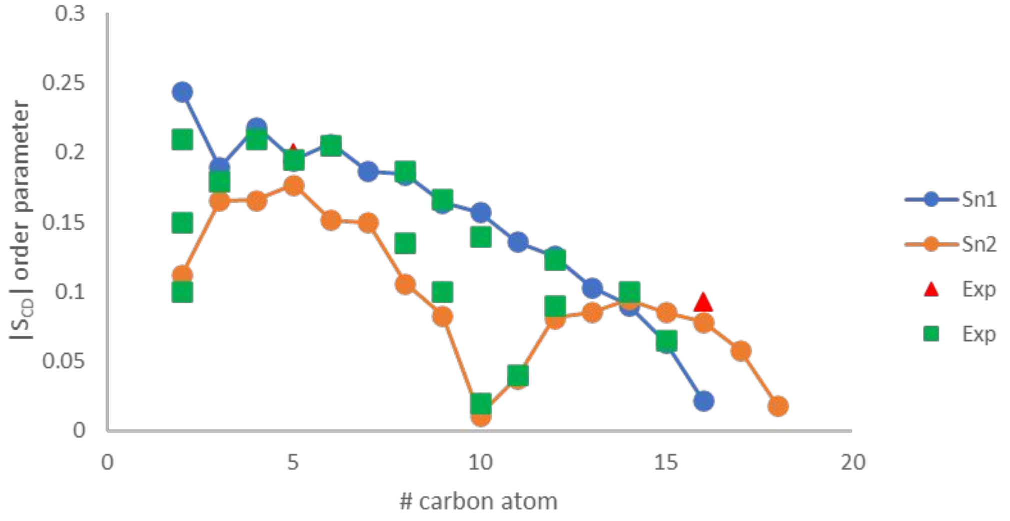

Figure S2. Calculated S_{CD} order parameters for POPC compared to experimental values.[9,10]

1.3 DOPC structural properties

Table S3. Calculated bilayer structural properties for DOPC compared to experimental values.

DOPC	Area per lipid (Å^2)	Area compressibility modulus (mNm^{-1})	Volume per lipid (Å^3)	Thickness (Å)	Lateral lipid diffusion (10^{-8}cm^{2}s^{-1})
Calculated	69.77 ± 0.2	302.9 ± 12.0	1252 ± 1.1	36.5 ± 0.2	11± 0.3
Experiment	67.4, 72.5[11]	265, 318[12]	1303[12]	35.3-37.1[11]	11.5[8]

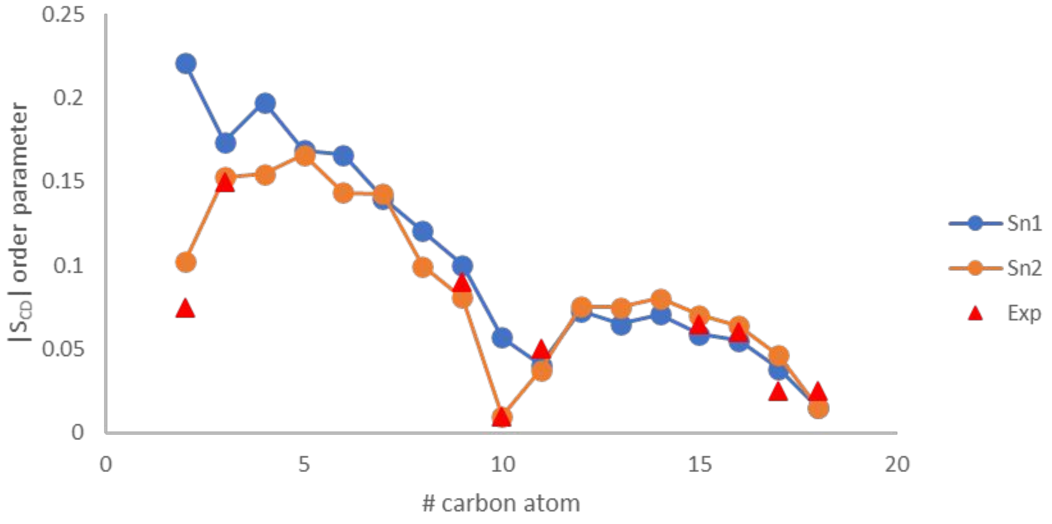

Figure S3. Calculated S_{CD} order parameters for DOPC compared to experimental values.[13]

1.4 DPPC structural properties

Table S4. Calculated bilayer structural properties for DPPC compared to experimental values.

DPPC	Area per lipid (Å^2)	Area compressibility modulus (mNm^{-1})	Volume per lipid (Å^3)	Thickness (Å)	Lateral lipid diffusion (10^{-8}cm^{2}s^{-1})
Calculated	62.9 ± 0.3	284.7 ± 10.7	1227 ± 0.2	38.2 ± 0.1	11.5 ± 0.5
Experiment	63.1[14]	231[15]	1232[12]	38, 38.3[11]	12.5[16]

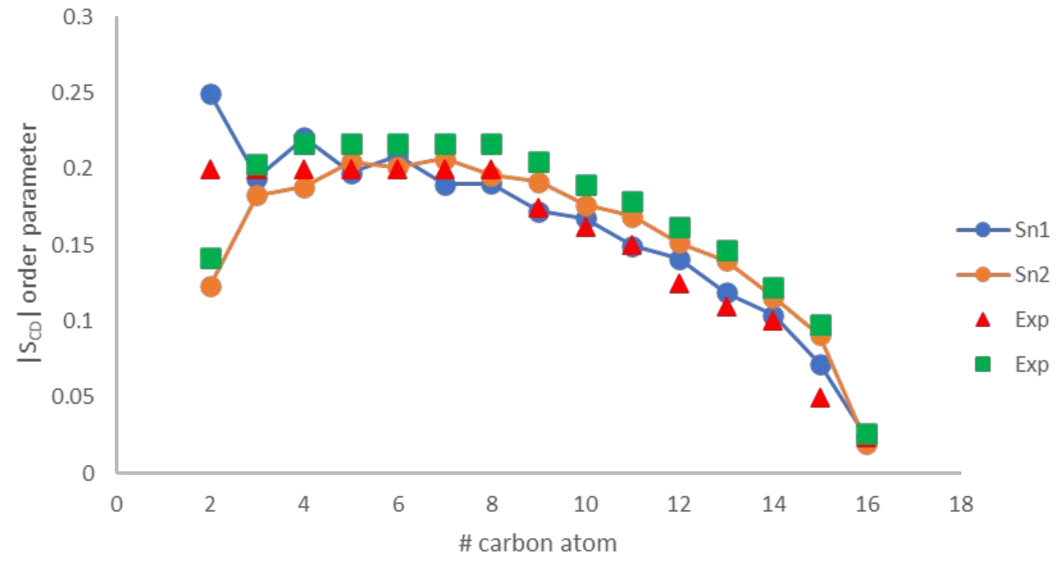

Figure S4. Calculated S_{CD} order parameters for DPPC compared to experimental values.[4,5]

2. Pure bilayer area per lipid over time

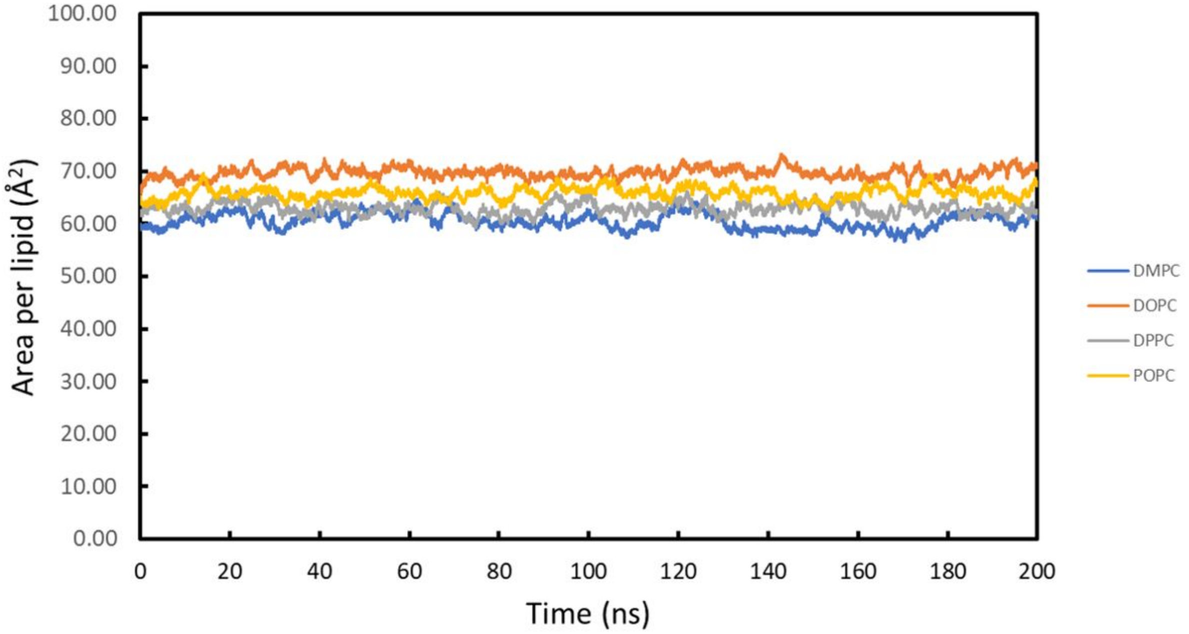

Figure S5. Area per lipid time-series for each bilayer over 200 ns.

3. Umbrella sampling histogram analysis

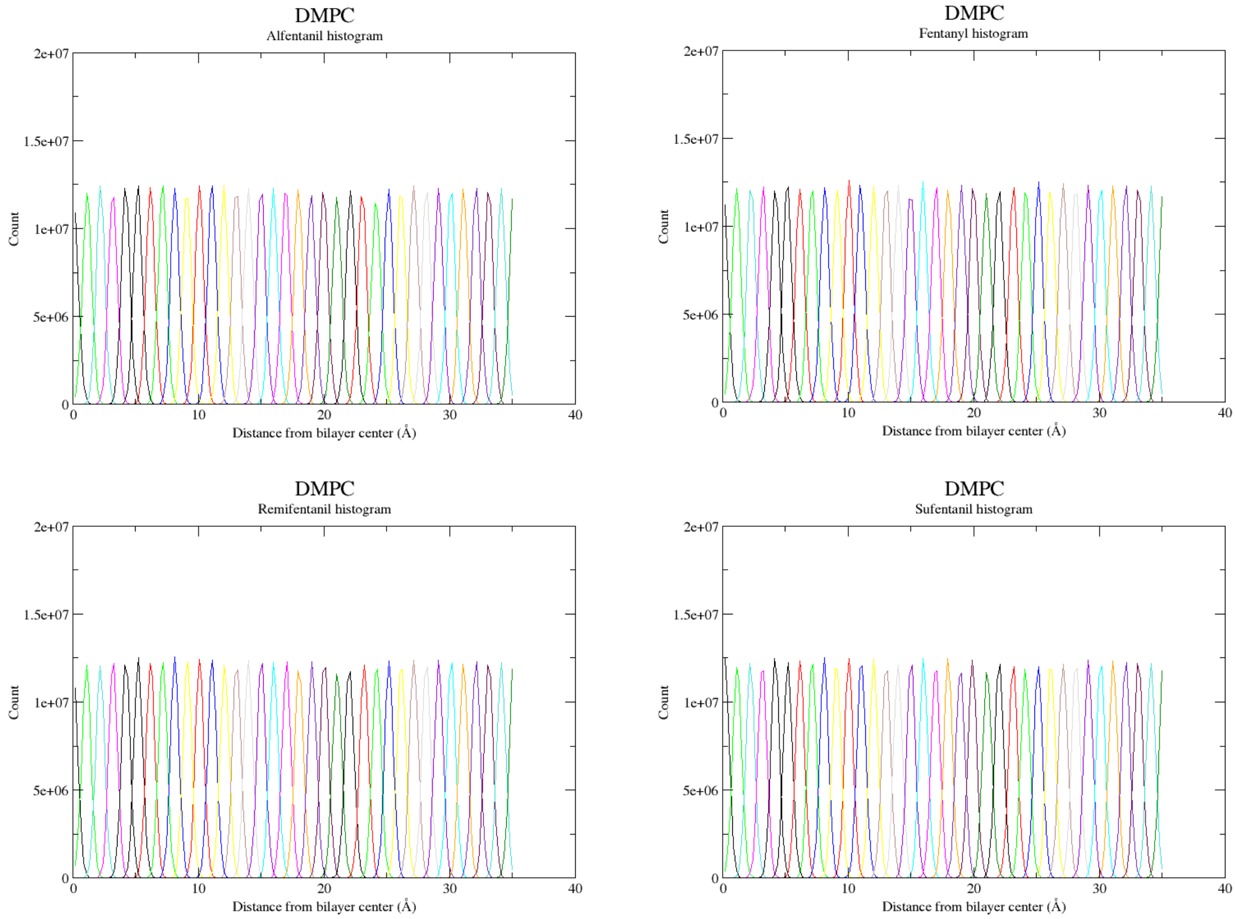

Figure S6. Umbrella sampling histograms for all drug molecules in the DMPC bilayer.

4. PMF, Z-diffusion and Z-resistance plots for each bilayer

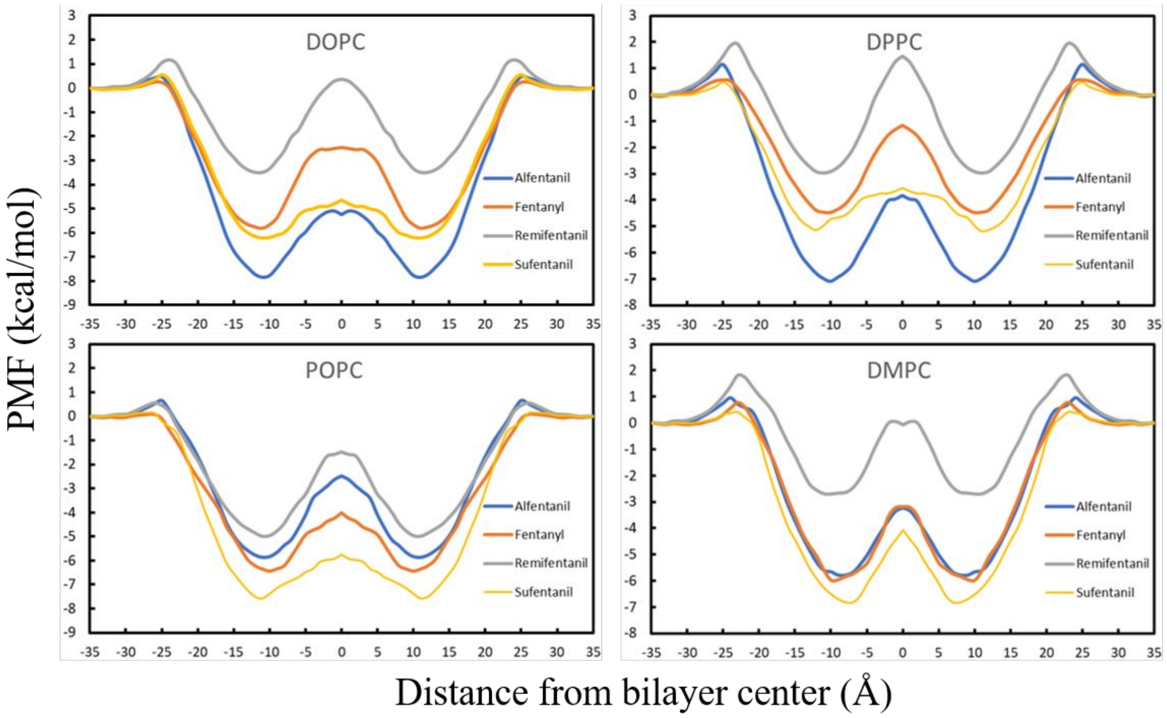

Figure S7. Free energy profiles calculated for all permeating molecules in each bilayer

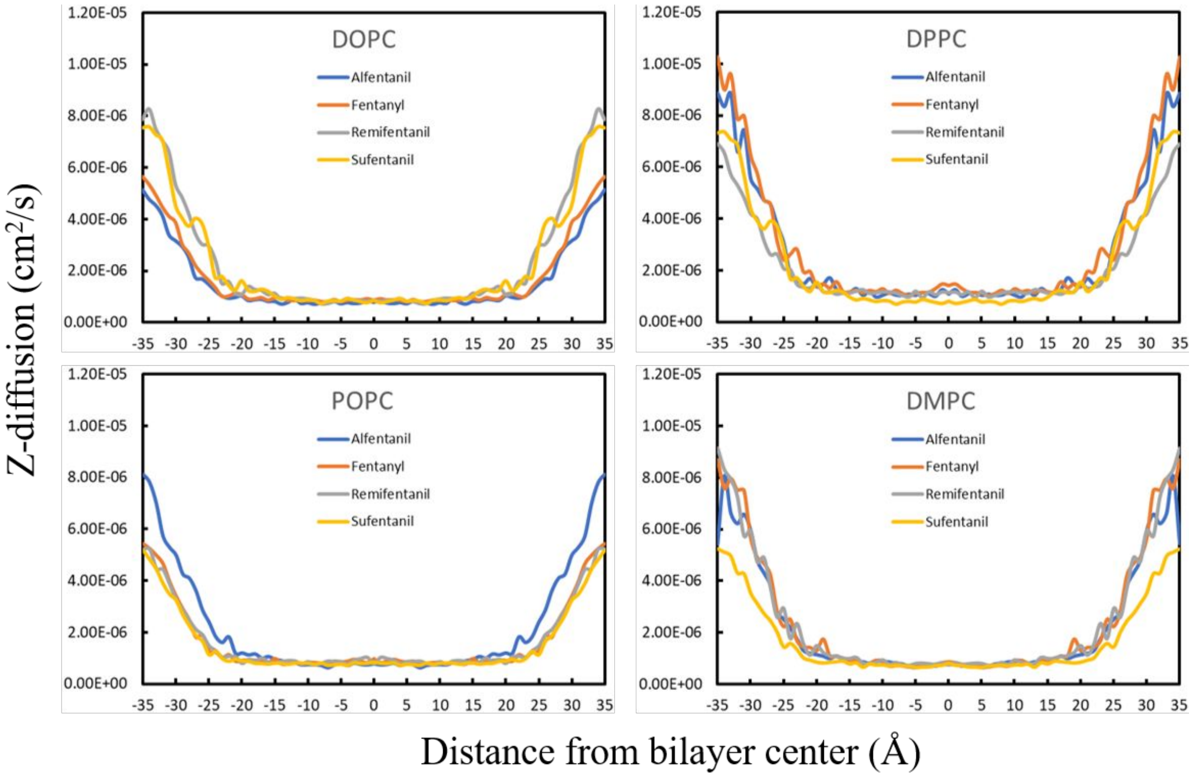

Figure S8. Z-diffusion profiles calculated for all permeating molecules in each bilayer

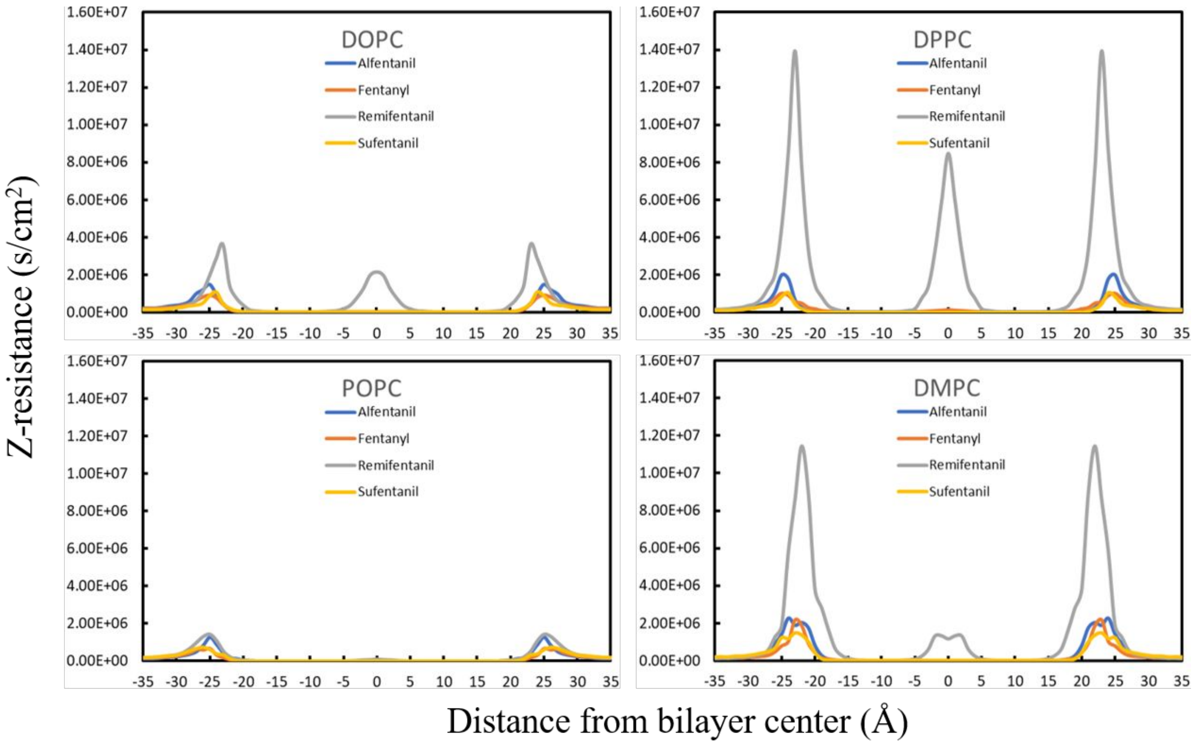

Figure S9. Z-resistance profiles calculated for all permeating molecules in each bilayer

References

(1) Kučerka, N.; Liu, Y.; Chu, N.; Petrache, H. I.; Tristram-Nagle, S.; Nagle, J. F. Structure of Fully Hydrated Fluid Phase DMPC and DLPC Lipid Bilayers Using X-Ray Scattering from Oriented Multilamellar Arrays and from Unilamellar Vesicles. *Biophys. J.* **2005**, *88* (4), 2626–2637. https://doi.org/10.1529/biophysj.104.056606.

(2) Rawicz, W.; Olbrich, K. C.; McIntosh, T.; Needham, D.; Evans, E. A. Effect of Chain Length and Unsaturation on Elasticity of Lipid Bilayers. *Biophys. J.* **2000**, *79* (1), 328–339. https://doi.org/10.1016/S0006-3495(00)76295-3.

(3) Almeida, P. F. F.; Thompson, T. E. Lateral Diffusion in the Liquid Phases of Dimyristoylphosphatidylcholine/Cholesterol Lipid Bilayers : A Free Volume Analysis. *Biochemistry* **1992**, *31* (29), 6739–6747. https://doi.org/10.1021/bi00144a013.

(4) Petrache, H. I.; Dodd, S. W.; Brown, M. F. Area per Lipid and Acyl Length Distributions in Fluid Phosphatidylcholines Determined by 2H NMR Spectroscopy. *Biophys. J.* **2000**, *79* (6), 3172–3192. https://doi.org/10.1016/S0006-3495(00)76551-9.

(5) Douliez, J. P.; Léonard, A.; Dufourc, E. J. Restatement of Order Parameters in Biomembranes: Calculation of C-C Bond Order Parameters from C-D Quadrupolar Splittings. *Biophys. J.* **1995**, *68* (5), 1727–1739. https://doi.org/10.1016/S0006-3495(95)80350-4.

(6) Kučerka, N.; Tristram-Nagle, S.; Nagle, J. F. Structure of Fully Hydrated Fluid Phase Lipid Bilayers with Monounsaturated Chains. *J. Membr. Biol.* **2006**, *208* (3), 193–202. https://doi.org/10.1007/s00232-005-7006-8.

(7) Binder, H.; Gawrisch, K. Effect of Unsaturated Lipid Chains on Dimensions, Molecular Order and Hydration of Membranes. *J. Phys. Chem. B* **2001**, *105* (49),

12378–12390. https://doi.org/10.1021/jp010118h.

(8) Filippov, A.; Orädd, G.; Lindblom, G. Influence of Cholesterol and Water Content on Phospholipid Lateral Diffusion in Bilayers. *Langmuir* **2003**, *19* (16), 6397–6400. https://doi.org/10.1021/la034222x.

(9) Seelig, J.; Waespe-Šarĉević, N. Molecular Order in Cis and Trans Unsaturated Phospholipid Bilayers†. *Biochemistry* **1978**, *17* (16), 3310–3315. https://doi.org/10.1021/bi00609a021.

(10) Perly, B.; Smith, I. C. P.; Jarrell, H. C. Acyl Chain Dynamics of Phosphatidylethanolamines Containing Oleic Acid and Dihydrosterculic Acid: 2H NMR Relaxation Studies. *Biochemistry* **1985**, *24* (17), 4659–4665. https://doi.org/10.1021/bi00338a027.

(11) Kučerka, N.; Nagle, J. F.; Sachs, J. N.; Feller, S. E.; Pencer, J.; Jackson, A.; Katsaras, J. Lipid Bilayer Structure Determined by the Simultaneous Analysis of Neutron and X-Ray Scattering Data. *Biophys. J.* **2008**, *95* (5), 2356–2367. https://doi.org/10.1529/biophysj.108.132662.

(12) Evans, E.; Rawicz, W.; Smith, B. A. Concluding Remarks Back to the Future: Mechanics and Thermodynamics of Lipid Biomembranes. *Faraday Discussions*. Royal Society of Chemistry December 10, 2012, pp 591–611. https://doi.org/10.1039/c2fd20127e.

(13) Warschawski, D. E.; Devaux, P. F. Order Parameters of Unsaturated Phospholipids in Membranes and the Effect of Cholesterol: A 1H-13C Solid-State NMR Study at Natural Abundance. *Eur. Biophys. J.* **2005**, *34* (8), 987–996. https://doi.org/10.1007/s00249-005-0482-z.

(14) Kučerka, N.; Tristram-Nagle, S.; Nagle, J. F. Closer Look at Structure of Fully Hydrated Fluid Phase DPPC Bilayers. *Biophys. J.* **2006**, *90* (11), L83–L85. https://doi.org/10.1529/biophysj.106.086017.

(15) Nagle, J. F.; Tristram-Nagle, S. Structure of Lipid Bilayers. *Biochimica et Biophysica Acta - Reviews on Biomembranes*. Elsevier November 10, 2000, pp 159–195. https://doi.org/10.1016/S0304-4157(00)00016-2.

(16) Scheldt, H. A.; Huster, D.; Gawrisch, K. Diffusion of Cholesterol and Its Precursors in Lipid Membranes Studied by 1H Pulsed Field Gradient Magic Angle Spinning NMR. *Biophys. J.* **2005**, *89* (4), 2504–2512. https://doi.org/10.1529/biophysj.105.062018.
